# Does a Hot Drink Provide Faster Absorption of Paracetamol Than a Tablet? A Pharmacoscintigraphic Study in Healthy Male Volunteers

**DOI:** 10.1007/s11095-014-1309-3

**Published:** 2014-02-21

**Authors:** Lee Ann Hodges, Alison Hughes, Darren Targett, Michael J. Durcan

**Affiliations:** 1 Bio-Images Research Ltd, Within Glasgow Royal Infirmary, 84 Castle Street, Glasgow, G4 0SF UK; 2 GlaxoSmithKline Consumer Healthcare, St George’s Avenue Weybridge, Surrey, KT13 0DE UK

**Keywords:** gamma scintigraphy, gastric emptying, paracetamol, pharmacokinetics

## Abstract

**Purpose:**

To investigate the hypothesis that paracetamol is absorbed faster from a hot drink than from a standard tablet using simultaneous scintigraphic imaging and pharmacokinetic sampling.

**Methods:**

Twenty-five healthy male volunteers received both paracetamol formulations in a randomised manner. The formulation administered in the first treatment arm was radiolabelled to allow scintigraphic monitoring. In both treatment arms, blood samples were taken for assessing paracetamol absorption.

**Results:**

Following the hot drink, paracetamol absorption was both significantly faster and greater over the first 60 min post-dose compared with the tablet, as evidenced by the median time to reach t_0.25_ μg/mL of 4.6 and 23.1 min, respectively, and AUC_0-60_ of 4668.00 and 1331.17 h*ng/mL, respectively. In addition, t_max_ was significantly shorter for the hot drink (median time = 1.50 h) compared with the tablet (1.99 h). However, C_max_ was significantly greater following the tablet (9,077 ng/mL) compared with the hot drink (8,062 ng/mL). Onset of gastric emptying after the hot drink was significantly faster than after the standard tablet (7.9 *versus* 54.2 min), as confirmed scintigraphically.

**Conclusions:**

Compared with a standard tablet, a hot drink provides faster absorption of paracetamol potentially due to more rapid gastric emptying.

## INTRODUCTION

The common cold is one of the most frequent human illnesses worldwide (1) and, although no cure exists, symptoms are treatable. A plethora of cold remedies exist but few have proven effectiveness, although paracetamol has shown greater effectiveness than placebo in treating symptoms associated with upper respiratory tract infection, including sore throat (2), headache (3) and fever (4). Cold remedies are available in a variety of formats, including hot drink and tablet forms. However, there have been very few clinical studies conducted to investigate their potential for rapid symptom control. Whilst capsules and tablets are more convenient for many customers, hot drink remedies are associated with greater comfort and they provide active ingredients in solution that may result in them reaching the bloodstream and being bioavailable faster than tablet formulations. Previous data from a smaller study of the absorption of paracetamol from a hot drink formulation (although not specifically designed to estimate pharmacokinetic parameters) indicated that the paracetamol from a hot drink was absorbed more quickly than historically seen with a solid dose formulation (5).

Absorption of paracetamol from the stomach is negligible but is rapid and significant from the small intestine (6), making rapid gastric emptying a key approach to reducing the delay between drug ingestion and onset of symptom control. Fast-dissolving tablets have been shown to empty from the stomach more quickly than standard tablets, resulting in earlier appearance of the drug in the plasma (7–9) and most importantly, more rapid pain relief (10). Previous studies using the dual investigative techniques of gamma scintigraphic imaging combined with concurrent pharmacokinetic (PK) assessment have shown that the rate of gastric emptying is directly proportional to the rate of paracetamol absorption (7, 8). Since paracetamol is more soluble in hot water but only sparingly soluble in cold water, it is hypothesised that presenting paracetamol as a hot drink will potentially increase the rate of gastric emptying of the drug as it is already in solution form, negating the requirement for prior disintegration and dissolution of conventional tablets.

This clinical study was designed to compare the *in vivo* behaviour of two paracetamol formulations: one a hot drink and the other as a standard tablet. Although the hot drink contained additional ingredients of phenylephrine and ascorbic acid (phenylephrine is commonly used as a nasal decongestant to help relieve a blocked nose and ascorbic acid [Vitamin C] is a common ingredient of cold and flu remedies) the pharmacology of these ingredients does not suggest that any effect on the PK of paracetamol is likely.

The simultaneous monitoring of formulation behaviour using gamma scintigraphy and blood sampling for PK analysis was utilised to establish the link between formulation disintegration and gastric emptying with resultant serum concentrations of paracetamol. This study fills a knowledge gap where previously there were no data on the transit rates of hot drink formulations through the gastrointestinal (GI) tract.

The primary objective of this healthy volunteer study was to investigate whether paracetamol in a hot drink reaches the plasma faster than from standard tablets, as determined by the time to reach a plasma concentration of 0.25 μg/mL (t_0.25_ μg/mL). Other indicators of the speed of early paracetamol absorption included AUC_0-30_, AUC_0-60_, t_max_ and C_max_. The use of these concentrations to determine the PK parameters was standard and the lower limit of quantification (LLOQ) was 0.05 μg/mL, so it was proposed that five times the LLOQ was a robust indicator of paracetamol presence in the blood. The scintigraphic data provided information on the *in vivo* fate of both formulations to allow a correlation to be made between PK parameters and gastric emptying and disintegration profiles.

## MATERIALS AND METHODS

### Materials

Hot drink sachets (Beechams Flu Plus Hot Lemon Sachets) and standard paracetamol tablets (Panadol Original Tablets) were supplied by the Clinical Supplies Department, GlaxoSmithKline Consumer Healthcare UK. Both products were obtained from a commercially available batch and packaged in commercial packaging. The paracetamol dose was the same for both the tablet and hot drink formulations (1,000 mg). Technetium-99 m diethylenetriamine pentaacetic acid (^99m^Tc-DTPA) was provided by the West of Scotland Radionuclide Dispensary, Glasgow, UK. Lactose monohydrate for radiolabelling procedures was obtained from DMV-Fonterra, The Netherlands.

### Formulation Radiolabelling

#### Hot Drink

A volume of ^99m^Tc-DTPA sufficient to provide approximately 3.8 MBq at the target dosing time was added to 150 mL of hot water. The contents of the sachet were mixed with this radiolabelled water. The hot drink was allowed to cool sufficiently to be drinkable and was at a temperature between 48 and 50°C at time of dosing.

#### Standard Paracetamol Tablets

Radiolabelled lactose monohydrate was prepared by mixing lactose monohydrate with a volume of ^99m^Tc-DTPA sufficient to provide approximately 1.9 MBq per tablet at the target dosing time, following drying in hot air. The tablets were drilled to a fixed depth using a microdrill then filled with the required dose of radiolabelled lactose monohydrate (approximately 5 mg) and sealed with a small amount of bone cement. This previously validated radiolabelling methodology has been used in other scintigraphic studies (11, 12). Unpublished data from work previously conducted within this clinical centre confirmed that the complete release of radiolabel correlated well with complete tablet disintegration.

Two 500 mg tablets were given orally with 150 mL water at room temperature.

### Clinical Study

#### Study Design

This was a phase IV, single centre, open-label, randomised, two-way crossover study conducted in healthy male volunteers. The study was performed according to the protocol and in accordance with the guidelines of the Declaration of Helsinki and Good Clinical Practice (GCP). The protocol and relevant study documentation were approved by the Scotland A Research Ethics Committee. The Administration of Radioactive Substance Advisory Committee (ARSAC) approved the radiation dosimetry.

The following study treatments were administered in a randomised manner based on a Williams Latin Square design:

- Hot drink i.e. 1,000 mg paracetamol, 10 mg phenylephrine and 40 mg ascorbic acid prepared with 150 mL hot water
- Standard paracetamol tablets i.e. 2 × 500 mg tablets taken with 150 mL water at room temperature

To minimise radiation exposure to the subjects, only the formulation administered on the first dosing occasion was radiolabelled.

#### Study Population

A total of 25 healthy male volunteers were enrolled into the study. They provided written informed consent prior to participation in any study-specific investigations and underwent a screening medical investigation to ensure compliance with study criteria. The study population included non-smokers who were in good general health with a body mass index (BMI) in the range 18.0–29.9 kg/m^3^.

In addition, it was essential that the subjects did not suffer from any GI disorders that could impact on the expected ‘normal’ behaviour of the formulations following administration. As such, subjects with diabetes and current sufferers of migraine were excluded as they have been found to have altered gastric emptying (13, 14). Vegetarians were also excluded as there is evidence that paracetamol absorption is impaired in this population (15) and due to the standard meals provided on study assessment days. Female subjects were excluded due to the need for exposure to radiation and it has been observed that the menstrual cycle has been associated with changes in gastric emptying patterns (16). Subjects with egg allergy were also excluded due to the contents of the standardised breakfast, and any subjects with a BMI of ≥30 kg/m^2^ were excluded as shielding caused by bone, muscle, other organs and soft tissue can attenuate radioactive counts.

#### Study Procedures

Eligible subjects attended the study centre on two dosing occasions. On arrival at the study centre, subjects were questioned on adherence to study restrictions, which included pre-breakfast fasting of at least 10 h of which the final 2 h required abstinence from fluids as well. In the 72 h prior to dosing, subjects were not allowed any alcohol. They were also restricted from consuming any caffeine- or xanthine-containing beverages or foods and undertaking any strenuous physical activity in the 24 h prior to dosing. Food and fluid intake on the study days were monitored by study staff and consisted only of standard meals supplied. Subjects were also required to abstain from prescribed and over-the-counter medications for 14 days and 48 h pre-dose, respectively, unless the medication was approved by a study physician.

At 2 h pre-dose, subjects consumed a standard breakfast which comprised one scrambled egg, one slice of bacon, one slice of toast with 15 g butter and 5 g jam, 100 g hash browns and 200 mL whole milk. The consumption of this meal at this time was to enable the dosing of study treatments to occur in the ‘semi-fed’ state, which mimics the normal directions for usage of analgesic products.

Approximately 15–30 min pre-dose, a blood sample was taken and, on the first dosing occasion only, external radioactive markers (approximately 0.01 MBq ^99m^Tc) were taped to the chest and back to enable accurate alignment of sequential images. At the target dosing time, the subjects were given the study treatment and were required to complete dosing within 20 s.

The investigator (or designee) collected blood samples from an indwelling cannula placed in the subject’s arm at the following times: pre-dosing, then 3, 5, 7, 9, 11, 15, 20, 30, 45, 90, 120 and 180 min post-dosing to allow an assessment of paracetamol PK. The total blood volume taken at each timepoint was approximately 4 mL. The actual sample times were recorded alongside the nominal times on the Case Report Form (CRF). An acceptable blood sampling time was considered ± 30 s for up to 11 min, ± 1 min for 15, 20, 30 min, then ± 2 min from 30 min onwards. The total amount of blood removed during the two treatment visits for PK analysis was approximate to 104 mL. These blood samples were centrifuged and plasma fractions removed and frozen until shipping to a GSK-approved laboratory for analysis.

On the first dosing occasion only, scintigraphic images of 25 s duration each were taken from both anterior and posterior aspects immediately after dosing then every 5 min for a period of 15 min, then every 15 min to 2 h post-dose, every 20 min to 4 h post-dose and hourly to a maximum of 10 h post-dose. An acceptable scintigraphic imaging time was considered ± 2 min throughout the imaging period. The images were acquired using a Siemens eCam gamma camera with a 53.3 cm field of view and fitted with a low energy, high resolution collimator. Imaging was stopped once complete gastric emptying and release of radiolabel from the tablet (if applicable) was confirmed.

### Analytical Methods

#### Scintigraphy

Images were collected using the eSoft image acquisition software and subsequently analysed using the WebLink software.

The following parameters were derived from the analysis:

- Time to onset and completion of gastric emptying of a hot drink and standard paracetamol tablets
- Time and site of onset and complete disintegration of standard paracetamol tablets

#### Pharmacokinetics

The primary PK variable was the time taken to reach a plasma paracetamol concentration of 0.25 μg/mL (t_0.25_). Secondary PK variables were plasma concentrations of paracetamol at each PK sampling point, AUC_0-30_, AUC_0-60_, C_max_ and t_max_. The PK parameters AUC_0-30_, AUC_0-60_, C_max_ and t_max_ were derived from the observed individual subject drug concentration *versus* time data using non-compartmental methods in WinNonlin® Professional Version 5.0.1 or higher.

#### Statistics

All statistical analyses were performed using SAS Version 9.2.

Time to onset and completion of gastric emptying were analysed using an ANOVA model appropriate for a parallel group design. The time and site of onset and complete disintegration of standard paracetamol tablets were summarised using descriptive statistics.

The t_0.25_ and t_max_ parameters were subjected to a non-parametric analysis as the assumptions of normality and homogeneity of variance were not satisfied. A series of Wilcoxon rank sum tests as described by Hills and Armitage (17) was conducted and the Hodges-Lehmann estimate of the median difference between treatments was presented with a corresponding 95% confidence interval (CI) according to the method described by Hodges and Lehmann (18).

The AUC and C_max_ parameters were transformed prior to analysis using a logarithmic transformation (natural log) and analysed using an ANOVA model including factors for sequence, period and treatment (as fixed effects) and subject within sequence (as a random effect). The difference in log-transformed means and associated 95% CIs were back-transformed (exponentiated). For individual subjects, if AUC could not be calculated due to an insufficient number of quantifiable concentrations, then AUC was set to missing for that subject. If there were fewer than 12 subjects per treatment group for which AUC could be calculated, then a formal statistical analysis of AUC was not performed.

### Assessment of Safety/Tolerability

Safety was assessed by physical examinations, electrocardiogram (ECG), vital signs, laboratory safety evaluations (blood biochemistry, haematology and urinalysis) and adverse event (AE) monitoring. Subjects were actively questioned on AEs before dosing, throughout the study day and at follow-up. AEs spontaneously reported by subjects were also noted.

## RESULTS

**Fig. 1:**
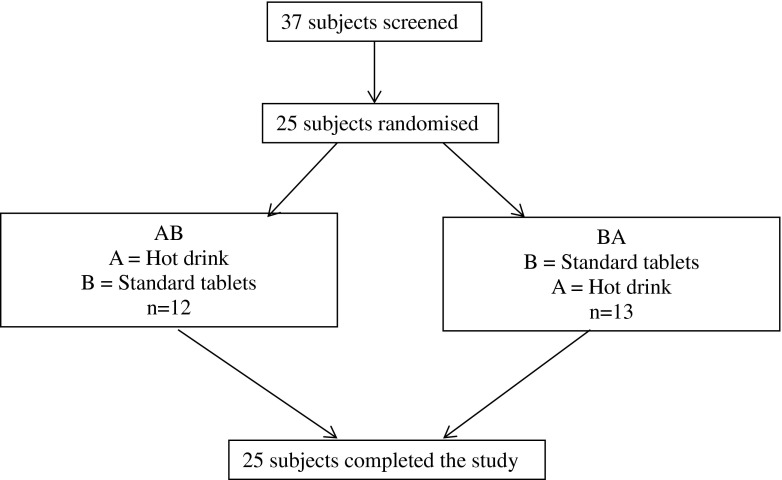
Subject disposition.

Of the 37 subjects screened, 25 were randomised and completed the study. A flow-chart showing the breakdown of subjects screened, randomised and treated is shown in Fig. 1.

The subjects had a mean (standard deviation [SD]) age of 30.5 (11.2) years (range: 18–51), a mean (SD) BMI of 24.74 (2.60) kg/m^2^ and all of the subjects were Caucasian.

The PK and scintigraphy analyses were performed on all subjects who were randomised, had any post-baseline PK or scintigraphic measurement, and had pre-dose plasma paracetamol concentration values of ≤100 ng/mL. One subject had a high pre-dose plasma paracetamol concentration value of 705 ng/mL in the first study period (where he was given the hot drink) and therefore was excluded from the analysis for this period only.

### Gamma Scintigraphy Results

**Fig. 2:**
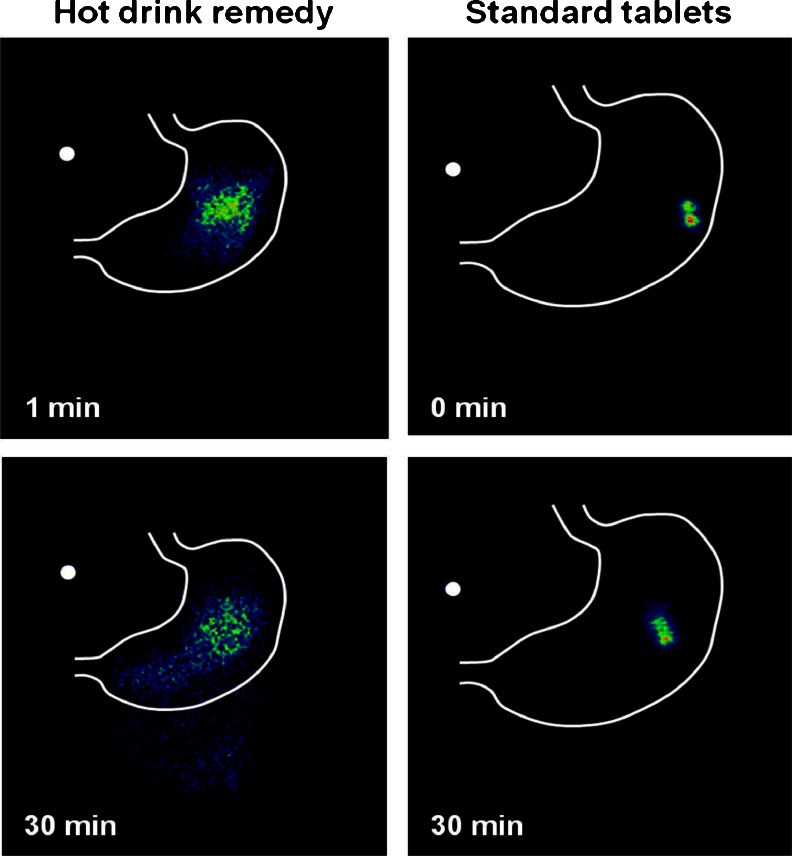
Example scintigraphic images of gastric emptying behaviour of hot drink and standard tablets (exact times recorded for each image taken).

Example scintigraphic images comparing the gastric emptying behaviour of the hot drink and the standard tablets are shown in Fig. 2. At 30 min post-dosing, images clearly indicated that the hot drink had commenced emptying into the small intestine while the tablets were still relatively intact. Results showed that the hot drink had a statistically faster onset of gastric emptying compared with the standard tablets, as observed from the adjusted mean onset times of 7.86 and 54.23 min, respectively (*p* < 0.0001) (Table I). However, there was no statistically significant treatment difference in time to complete gastric emptying, although the completion time was approximately 34 min faster for subjects dosed with the standard tablet due to the fact it started to empty later (Table I).

**Table I:** Times to Onset and Completion of Gastric Emptying

	Paracetamol hot drink (*N* = 11)	Standard paracetamol tablets (*N* = 13)	Difference (adjusted mean)	95% CI (*p* value)
	Adjusted mean (SE)	Adjusted mean (SE)		
Onset (minutes)	7.86 (3.91)	54.23 (3.60)	−46.37	−57.38, −35.35 (<0.0001)
Completion (minutes)	202.59 (16.81)	168.38 (15.46)	34.21	−13.16, 81.58 (0.15)

For all 13 subjects dosed with the radiolabelled standard tablets, disintegration of tablets commenced and completed in the stomach. It should be noted that some disintegration of the tablets would have occurred prior to observation of gastric emptying of the radiolabel since the radiolabel is centralised in the tablet core so some non-labelled disintegrated material will have been released prior to gastric emptying of the radiolabelled product. However, it has been shown that administration of radiolabelled and non-labelled paracetamol tablets (using the same method of radiolabelling as used in this study) have similar disintegration rates (8). Onset of disintegration occurred at 43 min (SD = 18.0) and completion occurred at 63 min (SD = 24.8) post-dosing.

### Pharmacokinetics Results

**Fig. 3:**
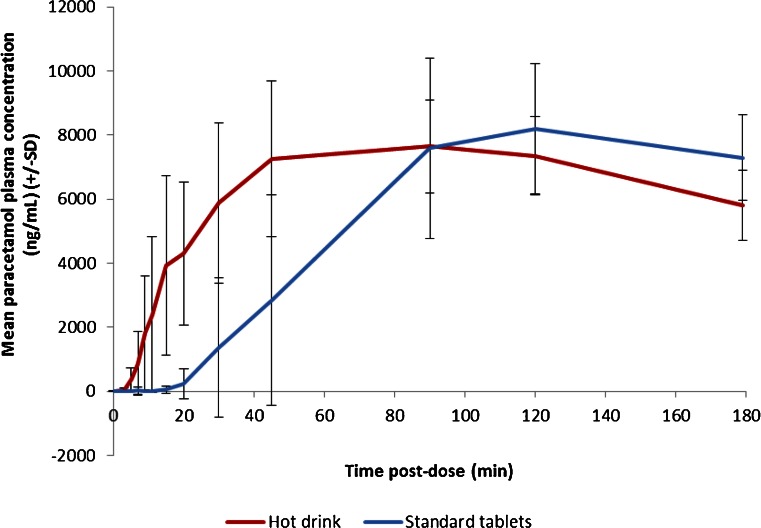
Mean paracetamol plasma concentration *vs.* time curves (*error bars* indicate between subject variability).

The mean plasma concentration *vs.* time profiles for both the hot drink and standard tablets are shown in Fig. 3. Appearance of paracetamol in the plasma was more rapid following administration of the hot drink when compared to the standard tablets. Tables II and III detail the PK parameters obtained and derived, as well as the results of the statistical treatments applied. The results demonstrated that t_0.25_ was significantly shorter in subjects dosed with the hot drink compared with standard paracetamol tablets, with median times to reach t_0.25_ of 4.59 and 23.14 min, respectively (*p* = 0.0004). The hot drink had a median t_max_ of 1.50 h which was significantly shorter than that of standard tablets (1.99 h) (*p*-value = 0.0058).

**Table II:** t_0.25_ and t_max_

	Paracetamol hot drink (*N* = 24)	Standard paracetamol tablets (*N* = 25)	95% CI (*p*-value)
	Median (range)	Median (range)	
t_0.25_ (minutes)	4.59 (3.4, 11.1)	23.14 (6.0, 46.6)	−27.31, −15.81 (0.0004)
t_max_ (hours)	1.50 (0.2, 2.0)	1.99 (0.7, 3.0)	−0.85, −0.33 (0.0058)

**Table III:** AUC and C_max_

	Paracetamol hot drink adjusted geometric mean	Standard paracetamol tablets adjusted geometric mean	95% CI (*p*-value)
AUC_0-30_ (hr*ng/mL)^a^	1313.69 (*n* = 24)	232.36 (*n* = 6)	N/A (N/A)
AUC_0-60_ (hr*ng/mL)^b^	4668.00 (*n* = 24)	1331.17 (*n* = 20)	2.39, 5.15 (<0.0001)
C_max_ (hr*ng/mL)^b^	8061.76 (*n* = 24)	9077.39 (*n* = 25)	0.82, 0.96 (0.0057)

^a^For AUC_0-30_, unadjusted geometric means are presented

^b^For AUC_0-60_ and C_max_, adjusted mean values calculated on log transformed data are back transformed and presented for each treatment group

AUC_0-30_ could only be calculated for 6 of the 25 subjects dosed with standard paracetamol tablets as there was insufficient quantifiable paracetamol plasma concentrations in the first 30 min for this treatment group. Since less than 12 subjects had this value calculated, statistical analyses were not performed for this parameter.

The adjusted geometric means for AUC_0-60_ for the hot drink and standard paracetamol tablets were 4668.00 and 1331.17 h*ng/mL respectively, indicating that paracetamol absorption over the first 60 min post-dose was statistically significantly greater with a hot drink compared with a standard paracetamol tablet (*p* < 0.0001). However, C_max_ was significantly higher for the standard paracetamol tablets compared with the hot drink. The adjusted geometric means were 9077.39 and 8061.76 ng/mL, respectively (*p* = 0.0057). These findings may be due to the fact that the hot drink provides a different bioavailability profile.

### Safety/Tolerability

There were no serious AEs (SAEs) or other significant AEs reported during the study and no subjects were discontinued due to AEs. The most commonly reported AE was haematuria in two subjects following the paracetamol hot drink, which was considered to be unrelated to the study product in both cases (in both cases mild haematuria was detected by dipstick and had resolved at the next assessment with no other associated problems reported). There were no significant safety issues with regard to vital signs, ECGs, or safety laboratory tests.

## DISCUSSION

Despite the vast array of cold remedies available, there have been very few clinical studies conducted to investigate their potential for rapid symptom control. Hot drink remedies are associated with providing greater comfort possibly because their intense taste helps stimulate the flow of saliva and mucus which lubricate and soothe the nose and throat, as well as helping to clear bacteria and viruses (19). Furthermore, the active ingredients are available in solution, with paracetamol being more soluble in hot water but only sparingly so in cold water, which may result in them reaching the bloodstream and being bioavailable faster than tablet formulations, thereby resulting in a quicker alleviation of discomfort. The premise that a hot drink would result in earlier paracetamol absorption in comparison to a standard tablet was based on previously published data that the rate of appearance of paracetamol in plasma correlated to the rate of gastric emptying of paracetamol. This is because paracetamol absorption depends on the rate of gastric emptying as it is absorbed in the small intestine rather than the stomach (20). A drug in solution will be emptied from the stomach faster (21) hence as the hot remedy is in solution, gastric emptying will be more rapid and absorption from the small intestine will occur sooner.

The current study evaluated the formulation behaviour and drug absorption behaviour of both hot drink and standard tablet formulations of paracetamol using simultaneous gamma scintigraphic imaging and blood sampling for PK analysis.

The data obtained clearly demonstrated the superiority of the hot drink over the standard tablet in achieving faster exposure of paracetamol, as observed from the median times to reach t_0.25_. Paracetamol absorption over the first 60 min post-dose was statistically significantly greater with a hot drink compared with that of a standard tablet. Furthermore, t_max_ was significantly shorter for the hot drink compared with standard paracetamol tablets. However, the C_max_ observed in the 3-h study period was significantly higher for the standard paracetamol tablets compared with the hot drink. However, it is important to note that total exposure (i.e. AUC_0-inf_) was not assessed in this study and therefore it is inappropriate to conclude that more paracetamol is being delivered with a hot drink compared to tablet formulation. It could perhaps be expected that the tablet produces a higher C_max_ compared to the hot drink formulation because the liquid hot drink is more spread out over the tissue at earlier timepoints and consequently there is a higher absorption rate per unit surface area, which results in C_max_ concentrations being higher at earlier timepoints. It is unlikely that temperature has a key effect on C_max_. The clinical significance of these PK differences on symptom relief remains to be fully elucidated and future large-scale studies may investigate this finding further, however, based on these results, it is proposed that a clinical benefit would be noted earlier following administration of a hot drink compared with a tablet.

In conjunction with the scintigraphic data that indicated that the time to onset of gastric emptying was significantly shorter for the hot drink, it can be inferred that the rapid drug absorption was a consequence of a more rapid onset of gastric emptying of the hot drink. Although the hot drink contained additional ingredients of phenylephrine and ascorbic acid, which might have been a contributing factor to the PK and gastric emptying differences, the pharmacology of phenylephrine and ascorbic acid does not suggest that this is likely.

This small-scale pilot study demonstrates interesting initial results, but further methodologically rigorous studies comprising large, long-term, prospective, randomised clinical trials are necessary to compare the absorption of different paracetamol formulations, together with further elucidation of the clinical significance of these differences on symptom relief.

## CONCLUSION

A hot drink of paracetamol has been shown to achieve faster and greater early drug absorption in comparison with a standard tablet formulation. Scintigraphic data supports the premise that more rapid gastric emptying of the hot drink contributed to the earlier appearance of paracetamol in the plasma. While comprehensive clinical data is not yet available to support the hypothesis that administering paracetamol in the form of a hot drink could result in more rapid alleviation of cold symptoms, results of this initial study allude to that potential.

## ABBREVIATIONS

AE(s): Adverse event(s)
ANOVA: Analysis of variance
ARSAC: Administration of radioactive substance advisory committee
AUC: Area under the curve
AUC_0-30_: Area under the concentration/time curve from 0 to 30 min
AUC_0-60_: Area under the concentration/time curve from 0 to 60 min
BMI: Body mass index
CI: Confidence interval
C_max_: Maximum concentration
CRF: Case Report Form
ECG: Electrocardiogram
GCP: Good clinical practice
GI: Gastrointestinal
LLOQ: Lower limit of quantification
PK: Pharmacokinetic
SAE: Serious adverse event
SAS: Statistical analysis software
SD: Standard deviation
t_0.25_: Time to reach 0.25mcg/mL
t_max_: Time at which C_max_ is observed
^99m^Tc DTPA: Technetium-99 m diethylenetriamine pentaacetic acid
